# Illinois State Dental Society

**Published:** 1895-06

**Authors:** Louis Ottofy

**Affiliations:** Masonic Temple, Chicago


### Illinois State Dental Society.

The thirty-first annual meeting of the Illinois State Dental Society was held at Galesburg, May 14 to 17, 1895. About 200 were in attendance. The following named persons were elected officers for the ensuing year: President, Walter A. Stevens, of Chicago; Vice-President, C. R. Taylor of Streator; Secretary, Louis Ottofy of Chicago; Treasurer, Edgar D. Swain of Chicago, Librarian, J. R. Rayburn of Fairbury; Chairman of Executive Committee, W. A. Johnston of Peoria. The next meeting will be held at Springfield, May 12 to 15, 1896.

LOUIS OTTOFY, Secretary.

Masonic Temple, Chicago.

